# Development and Validation of a Single-Tube Multiple-Locus Variable Number Tandem Repeat Analysis for *Klebsiella pneumoniae*

**DOI:** 10.1371/journal.pone.0091209

**Published:** 2014-03-10

**Authors:** Antoinette A. T. P. Brink, Christian J. H. von Wintersdorff, Christina F. M. van der Donk, Anne M. M. W. Peeters, Patrick S. Beisser, Ellen E. Stobberingh, Petra F. G. Wolffs

**Affiliations:** 1 Department of Medical Microbiology, Maastricht University Medical Center, Maastricht, The Netherlands; 2 School for Public Health and Primary Care, University of Maastricht, Maastricht, The Netherlands; Université d'Auvergne Clermont 1, France

## Abstract

Genotyping of *Klebsiella pneumoniae* is indispensable for management of nosocomial infections, monitoring of emerging strains –including extended-spectrum beta-lactamase (ESBL) producers-, and general epidemiology. Such objectives require a high-resolution genotyping method with a fixed scheme that allows (1) long-term retrospective and prospective assessment, (2) objective result readout and (3) library storage for database development and exchangeable results. We have developed a multiple-locus variable number tandem repeat analysis (MLVA) using a single-tube fluorescently primed multiplex PCR for 8 Variable Number Tandem Repeats (VNTRs) and automated fragment size analysis. The type allocation scheme was optimized using 224 *K. pneumoniae* clinical isolates, which yielded 101 MLVA types. The method was compared to the gold standard multilocus sequence typing (MLST) using a subset of these clinical isolates (n = 95) and found to be highly concordant, with at least as high a resolution but with considerably less hands-on time. Our results position this MLVA scheme as an appropriate, high-throughput and relatively low-cost tool for *K. pneumoniae* epidemiology.

## Introduction

*Klebsiella pneumoniae* is an opportunistic pathogen that can cause nosocomial infections such as pneumonia, urinary tract infections and bacteremia. Nosocomial *K. pneumoniae* strains often produce extended-spectrum beta-lactamase (ESBL). Typing of hospital-acquired strains is necessary to point out possible routes of transmission. For *K. pneumoniae*, a pulsed-field gel electrophoresis (PFGE) assay was described previously [1]. This is a sequence-independent method with a high resolution, but it is very laborious and does not allow interlaboratory comparison of results. A multilocus sequence typing (MLST) assay was described subsequently [2], which does provide portable data. However, the need for seven housekeeping gene PCRs per strain followed by two-sided sequence analysis may render this method too time-consuming for epidemiological analysis of large strain collections.

The *K. pneumoniae* genome contains variable numbers of tandem repeats (VNTR) as shown for many other types of bacteria [3]. VNTR analysis or multiple locus VNTR analysis (MLVA) is very useful for genotyping purposes and, like MLST, can provide portable results for interlaboratory comparison and database registration. Allele numbers are derived directly from the fragment sizes, and subsequently MLVA types are derived directly from the allele number combination [3], [4]. Possible interpretation variation that may arise from differences in fragment analysis platforms and/or software can be solved by providing sizing panels for each VNTR, to which users can calibrate their system.

A VNTR analysis scheme for typing of *K. pneumoniae* was reported recently [5]. However, in this study an agarose gel readout was used, which renders the method less suitable for high-throughput analysis. In addition, when small repeat units (e.g. 14 bp) are present, it is difficult to estimate the number of units from an agarose gel, necessitating additional sequence analysis. Capillary electrophoresis of fluorescently labeled VNTR PCR products allows accurate sizing and automated repeat number assignment without the need for sequence analysis, as shown previously in MLVA schemes for amongst others *Salmonella enterica* [6], *Staphylococcus aureus* [7] and *Escherichia coli* [8]. However, these schemes use two triplex or quadruplex PCRs to amplify the necessary VNTRs, while fluorescent PCR fragment analysis should allow the inclusion of many more PCR products in one reaction – as long as similarly sized products are differentially labelled. This was shown successfully for human short tandem repeats (STRs) analysis [9] where up to 16 loci are simultaneously amplified. In the present study, we aimed to develop an MLVA scheme for *K. pneumoniae* using eight VNTRs in one reaction. For this purpose, PCR primers were designed that allowed distinction of VNTRs based on either size or fluorescent label. The MLVA was developed using clinical *K. pneumoniae* strains. The MLVA was validated using MLST as the gold standard, and provided genotyping information with at least as high a resolution as MLST.

The MLVA scheme was used to investigate the genetic variability of ESBL-producing *K. pneumoniae* strains collected in our hospital between November 2005 and February 2013.

## Materials and Methods

### Bacterial isolates

To evaluate the variability of the selected VNTRs, we used a series of 12 clinical *K. pneumoniae* isolates (6 from the Democratic Republic of the Congo (DR Congo) and 6 from the Netherlands) representing 10 PFGE determined genotypes (data not shown), and the reference strains ATCC13883 [10] and ATCC700603 [11]. Subsequently, the MLVA scheme was evaluated using *K. pneumoniae* isolates collected in the department of Medical Microbiology, Maastricht University Medical Center: 54 non-ESBL producing isolates from urinary tract infections (UTIs), collected routinely between July 2011 and November 2011, and ESBL producing isolates from 130 patients, collected routinely between November 2005 and February 2013. Of 24 patients, more than one isolate was present, obtained within a 0–734 days' interval. In total, 224 clinical isolates were included in this study. In addition, a recently isolated OXA-48 positive strain that caused an outbreak in two Dutch hospitals was included [12]. An overview of the clinical strains is given in Table S1, including the time intervals with which additional samples were obtained from each of the 24 patients mentioned above. These additional strains were subjected to MLVA to determine the in vivo stability of the VNTRs. One of the strains (Strain 23 in Table S1) was subcultured for 20 consecutive days (1 colony picked and streaked onto a fresh agar plate) in order to determine the in vitro stability of the VNTRs.

For identification and antibiotic susceptibility testing, the Phoenix automated system (Beckton-Dickinson, Breda, The Netherlands) was used. ESBL production was confirmed phenotypically by combination disk diffusion tests [13].

### Identification of VNTR loci

At the start of the study, full genome sequences of three *K. pneumoniae* strains were known: the human pathogens MGH78578 [14] and NTUH-K2044 [15], and the N2-fixing broad host range endophytic *K. pneumoniae* strain 342 [16] (GenBank IDs: CP000647, AP006725 and CP000964, respectively). These reference strains were analyzed for the presence of tandem repeats using the Tandem Repeats Finder software, version 4.04 [17]. The maximum repeat unit size was set at 60 bp in order to avoid PCR product sizes exceeding 600 bp. This facilitates use of the standard size marker and standard fragment analysis module. VNTR loci that showed heterogeneity in repeat copy number *in silico* between at least two of the three reference strains were selected. Primers flanking the VNTR loci across an alignment of the three reference strains were designed using Primaclade software [18] and combined into primer pairs using Primer3Plus software [19]. VNTR PCR products obtained with the 14 evaluation strains were subjected to sequence analysis to determine their repeat composition and variability.

### Polymerase chain reaction (PCR)

Setup of PCR reactions was similar for MLST, VNTR single locus analysis and MLVA. Briefly, two colonies from an overnight culture were suspended in 500 µL sterile water, and this suspension was incubated at 95°C for 5 min. The lysate was centrifuged for 1 min at 10,000 rpm. Two μL of the supernatant was used in 20 µL PCR reactions containing 1× PCR buffer and 1 U HotStarTaq Plus polymerase (Qiagen, Venlo, The Netherlands), 200 µM each of dATP, dCTP, dGTP and dTTP (Roche, Almere, The Netherlands) and 4 pmol of each primer (Sigma-Aldrich, Zwijndrecht, The Netherlands; VIC-, NED- and PET-labelled primers from Applied Biosystems, Nieuwerkerk a/d IJssel, The Netherlands). The reactions were incubated for 5 min at 95°C to activate Taq polymerase. For VNTR PCR-sequencing and MLVA, the PCR program consisted of 30 cycles of 30 seconds at 94°C, 45 seconds 58°C and 60 seconds at 72°C. For MLVA, this was followed by 30 min 68°C to enhance 3′ adenosine addition by Taq polymerase and hence to prevent the formation of split peaks in fragment analysis. MLST was performed according to the scheme developed by Diancourt et al. [2]. PCR for ESBL genes were done according to previously published protocols for CTX-M [20], SHV and TEM [21]. Sequence analysis of PCR products was done using BigDye Terminator version 1.1, Applied Biosystems, according to the manufacturer's protocol.

### MLVA analysis

We aimed to include 8 VNTRs in one multiplex PCR reaction, allowing their distinction on an automated sequencer by the combination of product size and different fluorescent primer labels. Primer sequences are given in Table 1; the VNTRs in this table are sorted according to label and product size. The fluorescent label is always in the forward primer. The labeled PCR products were separated by capillary electrophoresis (fragment analysis) using the Applied Biosystems 3730 sequencer. The size standard used in capillary electrophoresis was LIZ600 (Applied Biosystems). Peak size analysis was performed using PeakScanner software (Applied Biosystems). Allele numbers were assigned based on the number of repeat units calculated to be present in each strain, starting by 0 for strains with the smallest possible hypothetical amplicon with flanking sequences only, 1 for strains with one repeat unit, etc. These allele definitions were entered into the MLVA plugin of the BioNumerics software (version 6.01, Applied Maths, Sint-Martens-Latem, Belgium) which was used to analyse the clinical strains. BioNumerics software was also used to calculate Diversity Indices for the different VNTRs, to generate band histograms in order to determine allele ‘bin’ sizes, and to draw minimum spanning trees. Clustering in minimum spanning trees was done using a categorical coefficient. A cluster was assigned when neighbouring types differed in no more than 1 VNTR locus and at least 5 isolates were present within the cluster.

**Table 1:** Variable Number Tandem Repeats selected for the MLVA scheme.

VNTR	Repeat unit size (bp)	Sequence		Coordinates^1^	Label	Product size range (bp)	Allele numbers	Index of diversity
52	21	Repeat	GACGGTAAACAGCCGCAGCAC (intergenic)					
		F^2^	TTTGGCGGCAGCGGTTTCCC	2128873–2128857	FAM	172–258	2–6	68.4
		R	GCCAGAAAAAGGCGCGCAGC	2128665–2128680				
45	12	Repeat	ACCTTGCTCGAT (intergenic; upstream of putative porin gene)					
		F	CGCTGACACATTGACGAAAACAGAGA	2924279–2924304	FAM	264–302	0–4	60.5
		R	ATGAATATTGCCCAGTTTCTGGAACAA	2924571–2924545				
53	11	Repeat	GCCGCTGCGCA (within gene encoding cell envelope integrity inner membrane protein TolA)					
		F	CGCAGAAGAAAGCGGAAG	834381–834398	FAM	315–367	0–5	44.1
		R	TGTTTTAGGCGCATTCTTACC	834724–834704				
51	3	Repeat	CGG (in putative lipoprotein gene *dedD*)					
		F	CCGCCGCGCCATCGTTAGAT	2974104–2974085	VIC	224–253	0–10	71.0
		R	TCAACGCGCCCAGCTGAACC	2973862–2973881				
60	7	Repeat	CGTGTGA (intergenic, immediately downstream of putative bacterial regulatory protein, MarR)					
		F	CGGTACGAATCTGTTGGATTAAG	2041248–2041270	VIC	311–391	0–11	68.9
		R	GGCCTTCTTCCGGGTCTAT	2041593–2041575				
10	57	Repeat	AAGAAACATCACAAAGCGGCCGCGAAACCGGCAGCCGAGCAGAAAGCGCAGGCGGCG (within putative acid shock protein gene *asr*)					
		F	AGCGCGCAGACGATGAGCAG	2198397–2198378	VIC	398–632	0–4	68.1
		R	AGCCCCGCAGTGGGGTTACT	2197938–2197957				
27	6	Repeat	CATGGT (within putative nickel/cobalt efflux protein gene *RcnA*)					
		F	CAGCGTCAGCGCCAGACCAA	465260–465279	NED	436–550	0–19	81.7
		R	CCATGGCCGGCCTGTGGTTT	465756–465737				
58	3	Repeat	GCA (within gene encoding penicillin-binding protein 1b)					
		F	CTATCTGGCGAACCAGACG	193771–193789	PET	501–538	0–10	57.0
		R	ATTATGACGGGCGATATAATAGGC	194294–194271				

1 All genomic coordinates given in this table refer to *K. pneumoniae strain* MGH78578 (GenBank ID: CP000647.1).

2 F and R refer to the primers used in the MLVA PCR.

## Results

### Development of *K. pneumoniae* MLVA

Using standard alignment parameters for repeat searching (matching base +2, mismatch -7, gap -7, minimum alignment score 50), Tandem Repeats Finder software detected >100 tandem repeats in the three *K. pneumoniae* full genome reference sequences. For repeats with a maximum unit size of 60 bp, *in silico* analysis of variability amongst the three reference genomes was performed. For repeats that had at least two variants among the three reference genomes, PCR primers were developed. The variability of these repeats amongst the 14 isolates in our evaluation set was assessed by PCR and sequence analysis. Ten VNTRs remained that showed at least three variants among the 14 isolates. Eight of these VNTRs allowed multiplexing into one MLVA PCR using fluorescently labelled forward primers. Any putative overlap in product size was corrected either by introduction of additional spacing between the primers or by using another fluorescent label (Table 1).

The 224 clinical *K. pneumoniae* isolates were then tested using the MLVA. Result files were imported in BioNumerics and the band histogram feature of the software was used to correct the predicted repeat unit bin sizes where necessary. Remarkably, for 3 VNTRs the repeat unit sizes as predicted *in silico* by Tandem Repeats Finder software were smaller when analysed *in vivo*: VNTR53, 51 and 27 were predicted to be 15, 12 and 18 bp respectively but actually were 11 bp, 3 and 6 bp. Sequence analysis sometimes revealed the presence of incomplete repeat units at the 5′ and 3′ boundaries of the total repeat, as was reported previously for *S. aureus* [7], and then the bin sizes were adjusted accordingly.

In total, the evaluation strains and the clinical strains together represented 101 MLVA types. The diversity indices per allele are given in Table 1; the diversity index for the MLVA as a whole was 96.8%.

### Stability of the VNTRs

The stability of the VNTRs under laboratory conditions was tested by subculturing one of the isolates for 20 consecutive days. All VNTRs remained the same size throughout the subculturing period (Repeat numbers 4-3-3-4-3-2-12-1), indicating that they were stable under normal laboratory conditions. Additional *K. pneumoniae* isolates obtained between 0–734 days after the original one were available from 24 patients, and all of these showed the same MLVA type as the original one (Table S1), indicating that the VNTRs are also stable under *in vivo* conditions. Interestingly, some of the additional isolates were ESBL producers whereas the original isolate was not.

### Comparison of MLVA and MLST

**Figure 1:**
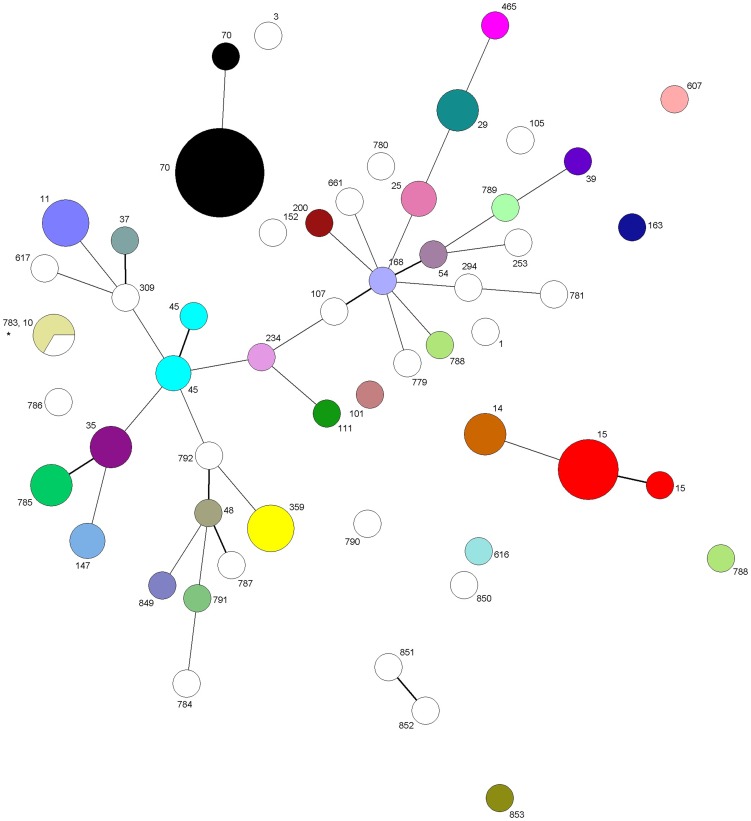
Minimum spanning tree of 95 isolates typed by MLVA and MLST. Each circle represents an MLVA type; the number of isolates within a given MLVA type is represented by the size of the circle. Single and double locus variants are connected by lines (short thick line and long thin line, respectively). Each color represents an ST; STs are also numbered. Because of the large number of STs in this study, some of the MLVA types containing only a single strain are left white.

MLST was performed on the 2 ATCC strains, 10/12 evaluation strains, 55/130 of the ESBL positive isolates and 28/54 prospectively collected UTI strains. These 95 isolates together represented 55 MLVA types and 52 sequence types (STs). A minimum spanning tree of the MLVA data, grouped by MLST data using colors (Figure 1) shows that both methods have a highly comparable typing resolution. In general, every MLVA type contains only one ST, except for one MLVA type that contains two STs (indicated by * in Figure 1). It should be noted here that ST783 differs from ST10 in only one silent mutation in the *pgi* gene. ST15, ST45 and ST70 are represented by 2 MLVA types each that differ in 1, 1 and 2 loci, respectively. The diversity index for MLST was 95.6%. The extra isolates from the 24 patients that we tested to determine the *in vivo* stability of the VNTRs were not included in the MLVA/MLST comparison nor in the determination of the diversity indices.

### ESBL production in MLVA complexes (MCs)

**Figure 2:**
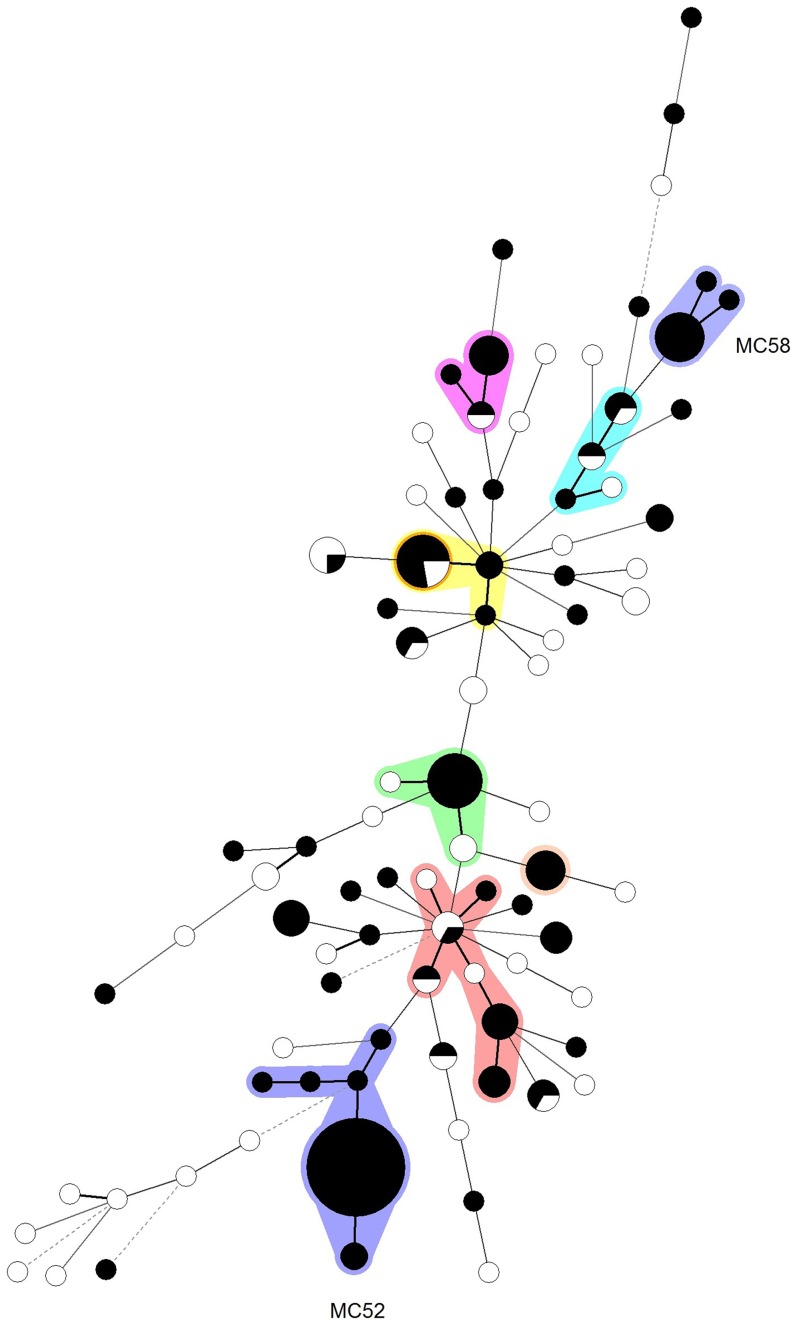
Minimum spanning tree of 184 unique *K. pneumoniae* strains typed by MLVA (130 ESBL producers and 54 non-ESBL producers). Colours surrounding more than one MLVA type indicate that these types belong to a cluster (MC). A cluster is assigned when neighbouring types differ in no more than 1 VNTR locus and at least 5 isolates are present within the cluster. ESBL producing strains are coloured black.

In Figure 2, a minimum spanning tree of all unique strains is given in which MLVA types are clustered using a categorical coefficient. The maximum difference between nodes in a cluster was one and the minimum number of isolates within a cluster was five. Although the current study group is restricted, and biased towards ESBL-producing isolates, several clusters of related MLVA types (MCs) were clearly observed. MCs 52 and 58 contain 100% ESBL-producing strains. The largest cluster was MC52 which contains only ESBL-producing strains. All strains from MC52 that were subjected to MLST (n = 17), were ST70. PCR and sequence analysis of beta-lactamase genes among these strains revealed that they all contained the *bla*_CTX-M15_ gene, in addition to wild-type TEM-1 and SHV-32. The latter is an SHV variant that is in fact a narrow-spectrum beta-lactamase [22]; hence it is the CTX-M15 that confers the ESBL phenotype in these strains.

MC58 contains 2 UTI strains from the prospectively collected series, 6 archival ESBL-producers and 2 archival strains from DR Congo. All strains from MC58 that were subjected to MLST (n = 8) were ST15.

### ESBL-producing MLVA types from year to year

**Figure 3:**
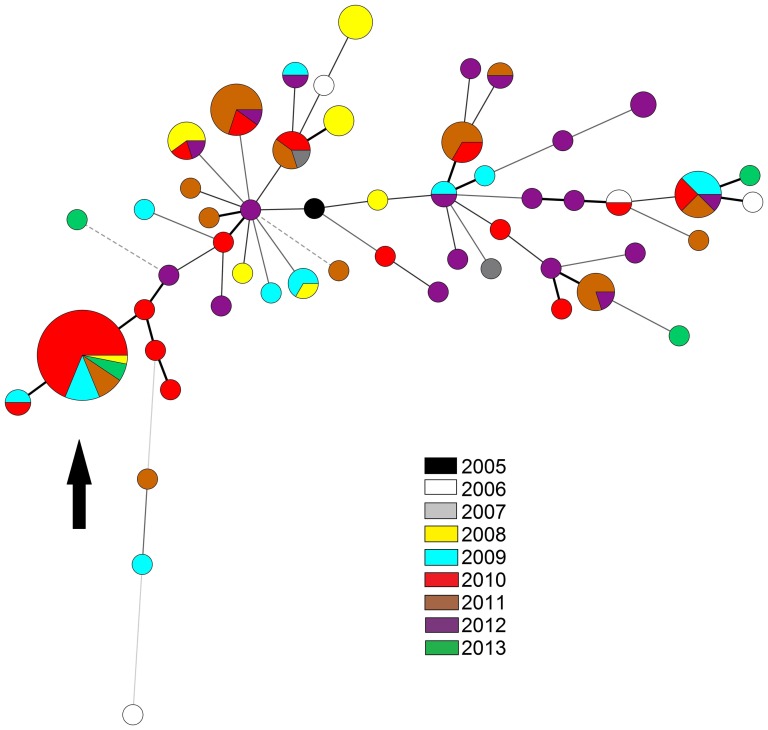
Minimum spanning tree of the 130 unique ESBL-producing *K. pneumoniae* strains collected in our hospital, typed by MLVA. Each color represents a year. MLVA type 72 is indicated by the black arrow. Thick line  =  single locus variant; thin line  =  double locus variant; dashed line  =  triple locus variant; dotted line  =  quadruple locus variant.

Figure 3 shows a minimum spanning tree of the MLVA types from all ESBL-producing isolates collected in our institute. The largest node represents MLVA type 72 (belonging to MLVA cluster MC52), which was first observed in 2008, increased in prevalence during 2009, peaked in 2010, decreased in 2011 and was not detected in 2012. Early 2013 however, this type was detected again: in a patient who had an infection with the same type back in early 2011, and in two new patients hospitalized in two different intensive care units. An epidemiological relation between these patients could not be detected.

The Dutch OXA-48 producing strain [12] showed an MLVA profile (5-3-3-4-4-3-12-1, MLVA type 88) that was observed in three other isolates (Table S1). These other isolates were all collected in 2008 and did not produce carbapenemases, hence the Dutch outbreak strain had not occurred in our hospital.

## Discussion

In this study we have developed an MLVA scheme for *K. pneumoniae* with a genotyping resolution equal to that of the gold standard MLST, but at considerably lower cost and hands-on time. The VNTRs in this MLVA scheme are stable under laboratory conditions and *in vivo*.

For VNTRs 53, 51 and 27 the repeat unit sizes *in vivo* were smaller than those predicted by Tandem Repeats Finder software. This is probably due to heterogeneity in the individual repeat units within one genome sequence, which leads to difficulties in recognition of the consensus sequence and hence in assignment of the correct repeat unit size.

A great advantage of our method is the fact that the PCR is done in a single reaction, which reduces the amount of disposables needed and greatly facilitates fragment analysis. Hands-on time depends on the lab automation level, but if one thermocycler and one fragment analysis system are present, 96 strains can comfortably be tested by MLVA within a day using one PCR plate and one capillary electrophoresis run. By contrast, MLST for 96 strains requires 7 PCR plates, 14 sequence reaction plates and 14 capillary electrophoresis runs. The analysis of the capillary data can also become faster with MLVA: in the present study we used PeakScan freeware for analysis of MLVA peaks where one has to review peaks visually, but it is also possible to use for example Applied Biosystems GeneMapper software or BioNumerics to automatically recognize peaks and assign the allele numbers. These differences in costs and hands-on time position MLVA as a useful tool for screening large collections of strains, or as a pre-screening tool that may be followed by MLST depending on the epidemiological question. For example, if typing data are to be correlated to previous literature describing the epidemiology of certain clones, MLST should certainly be included.

Because the maximum number of fluorescent labels in current generation fragment analysis methods is 5, one of which should be assigned to the size marker, we have used three FAM- and three VIC-labelled primers in the multiplex reaction. The strains used in this validation study all yielded products that could be assigned to a specific allele and no size overlap of products was observed – for example, the largest PCR product found for VNTR52 was still smaller than the smallest PCR product of the identically labelled VNTR45. It should be noted that “allele 0” for VNTR45 (no repeat units present, as calculated from the size of the amplicon) was detected only once in our test series. Its PCR product could easily be distinguished from the product of VNTR52 because of the difference in repeat unit size. Also among the other identically labelled VNTRs the repeat unit sizes are neither identical nor multiples of each other, which was designed with the intention to prevent mis-assignment of alleles. Should any future strains contain alleles that yield identically sized PCR products (e.g. because of incomplete repeats as described for *S. aureus* [7]), is it self-evident that the PCR can also be performed in two quadruplex reactions with four different labels each.

In general, the discriminatory power of our MLVA scheme may be underestimated not only because of the selection for ESBL-producing strains in this study, but also because most isolates were obtained within a single hospital. Nevertheless, this restricted data set yielded some interesting observations.

For example, the archival ESBL-producing strains from the 130 patients in this study represented 53 MLVA types. Of these 130 strains, 33 were MLVA type 72 (belonging to MLVA cluster 52) and had identical beta-lactamase gene profiles including the ESBL gene CTX M-15 and the narrow-spectrum beta-lactamase SHV-32. The first 11 isolates from this type were collected over a time span of 2 years, whereas the subsequent 15 isolates (including the four epidemiologically related patients) were collected within 3 months' time. However, for only four of the patients infected with this MLVA type an epidemiological relation could be determined (they had been in the same hospital ward within a three weeks' time interval). This type was not detected during the entire year 2012 but it was found again early 2013. These results suggest that strains with this MLVA type prevail among the general population but may be prone to a steep rise in their prevalence, indicating the need for prospective typing in hospital settings. Interestingly, the sequence type to which these strains belong (ST70) was described previously [23], but this previous study described a strain carrying a *bla*_KPC_, SHV-27 and no CTX-M type gene. These findings suggest that strains of this ST are highly capable of horizontal gene transfer.

It is important to notice that the number of strains belonging to an MC is restricted, but this number is likely to change when more strains will have been analysed and additional MCs will be discovered. We are currently developing a web-based application that allows users to enter the profiles they found, in order to provide unambiguous assignment of MLVA type numbers and to build a central database of all typed strains.

In conclusion, the MLVA method described in this paper is a promising tool for *K. pneumoniae* epidemiology and outbreak management – at least preceding or in combination with MLST. The analysis of additional strains will improve epidemiological insight, extend the number of MLVA profiles and MCs and reveal the true merit of this MLVA.

## Supporting Information

Table S1 Overview of all strains tested in this study, their ESBL status, MLVA profiles and Sequence Type.
(XLS)
